# (R)-NODAGA-PSMA: A Versatile Precursor for Radiometal Labeling and Nuclear Imaging of PSMA-Positive Tumors

**DOI:** 10.1371/journal.pone.0145755

**Published:** 2015-12-23

**Authors:** Eleni Gourni, Coline Canovas, Victor Goncalves, Franck Denat, Philipp T. Meyer, Helmut R. Maecke

**Affiliations:** 1 German Cancer Consortium (DKTK), Heidelberg, Germany; 2 Department of Nuclear Medicine, University Hospital Freiburg, Freiburg, Germany; 3 German Cancer Research Center (DKFZ), Heidelberg, Germany; 4 Institut de Chimie Moléculaire de l'Université de Bourgogne, UMR6302, CNRS, University Bourgogne Franche-Comté, Dijon, France; Monash University, AUSTRALIA

## Abstract

**Purpose:**

The present study aims at developing and evaluating an urea-based prostate specific membrane antigen (PSMA) inhibitor suitable for labeling with ^111^In for SPECT and intraoperative applications as well as ^68^Ga and ^64^Cu for PET imaging.

**Methods:**

The PSMA-based inhibitor-lysine-urea-glutamate-coupled to the spacer Phe-Phe-D-Lys(suberoyl) and functionalized with the enantiomerically pure prochelator (R)-1-(1-carboxy-3-carbotertbutoxypropyl)-4,7-carbotartbutoxymethyl)-1,4,7-triazacyclononane ((R)-NODAGA(tBu)_3_), to obtain (R)-NODAGA-Phe-Phe-D-Lys(suberoyl)-Lys-urea-Glu (CC34). CC34 was labeled with ^111^In, ^68^Ga and ^64^Cu. The radioconjugates were further evaluated in vitro and in vivo in LNCaP xenografts by biodistribution and PET studies. Biodistribution studies were also performed with ^68^Ga-HBED-CC-PSMA (HBED-CC: *N*,*N′*-bis[2-hydroxy-5-(carboxyethyl)benzyl]ethylenediamine-*N*,*N′*-diacetic acid) and ^111^In-PSMA-617 for comparison.

**Results:**

^68^Ga-CC34, ^64^Cu-CC34, and ^111^In-CC34 were prepared in radiochemical purity >95%. ^68/nat^Ga-CC34, ^64/nat^Cu-CC34, ^111/nat^In-CC34, ^68/nat^Ga-HBED-CC-PSMA, and ^111/nat^In-PSMA-617 exhibited high affinity for the LNCaP cells, with K_d_ values of 19.3±2.5 nM, 27.5±2.7 nM, 5.5±0.9 nM, 2.9±0.6 nM and 5.4±0.8 nM, respectively. They revealed comparable internalization profiles with approximately 75% of the total cell associated activity internalized after 3 h of incubation. ^68^Ga-CC34 showed very high stability after its administration in mice. Tumor uptake of ^68^Ga-CC34 (14.5±2.9% IA/g) in LNCaP xenografts at 1 h p.i. was comparable to ^68^Ga-HBED-CC-PSMA (15.8±1.4% IA/g) (*P* = 0.67). The tumor-to-normal tissue ratios at 1 and 2 h p.i of ^68^Ga-CC34 were also comparable to ^68^Ga-HBED-CC-PSMA (*P*>0.05). Tumor uptake of ^111^In-CC34 (28.5±2.6% IA/g) at 1 h p.i. was lower than ^111^In-PSMA-617 (52.1±6.5% IA/g) (*P* = 0.02). The acquisition of PET-images with ^64^Cu-CC34 at later time points showed wash-out from the kidneys, while tumor uptake still remained relatively high. This resulted in an increased tumor-to-kidney ratio over time.

**Conclusions:**

^68^Ga-CC34 is comparable to ^68^Ga-HBED-CC-PSMA in terms of tumor uptake and tumor to normal tissue ratios. ^64^Cu-CC34 could enable high contrast imaging of PSMA positive tissues characterized by elevated expression of PSMA or when delayed imaging is required. ^64^Cu-CC34 is currently being prepared for clinical translation.

## Introduction

Prostate cancer is the most common malignancy found in men and the second leading cause of cancer death in the US. In 2015, it is estimated that a total of 220,800 new prostate cancer cases will be diagnosed while 27,540 prostate cancer deaths are predicted to occur [1]. Therefore, efforts to discover and evaluate new diagnostic and therapeutic biomarkers for prostate cancer continue. Prostate specific membrane antigen (PSMA) is a well-established target for diagnostic and potential therapeutic applications. PSMA is a 750 amino acid type II integral membrane glycoprotein which is primarily expressed in healthy human prostate epithelium and in non-prostatic solid tumor vasculature without being shed into the circulation [2]. It is overexpressed by almost all prostate cancers with an increased expression by a factor of about 1000 in poorly differentiated, metastatic, and hormone-refractory cases [3–5]. The proposed pharmacophore for PSMA active site can be divided into three parts; three carboxylic groups, a carbonyl oxygen as part of the zinc complexation and nearby aromatic residues [6].

PSMA was originally targeted by the ^111^In-labeled monoclonal antibody 7E11-C5 (ProstaScint^®^; Cytogen Corporation, Princeton, NJ) which specifically binds to the PSMA+ human adenocarcinoma cell line LNCaP [7]. 7E11-C5 only binds to the intracellular site of PSMA (amino terminus) only accessible on necrotic tumors [8], therefore this tracer did lack wide acceptance in the field of nuclear medicine for the detection of prostate cancer. Radiolabeled monoclonal antibodies which bind to the extracellular site of PSMA were further developed [9–13]. Their successful preclinical evaluation and promising clinical assessment justified the utility of PSMA in the diagnosis and the potential therapy of prostate cancer [14,15]. As part of the ongoing efforts of several groups to develop new PSMA-specific ligands which outperform the disadvantages of antibodies such as inadequate pharmacokinetics and tissue accessibility, several chemical scaffolds such as PSMA inhibitors of low molecular weight have been synthesized and evaluated [16]. Among them, the urea-based PSMA inhibitors, functionalized to be used for imaging with Single-Photon Emission Computed Tomography (SPECT) or Positron Emission Tomography (PET), were able to successfully image PSMA-expressing xenografted mice [17–29].

**Fig 1:**
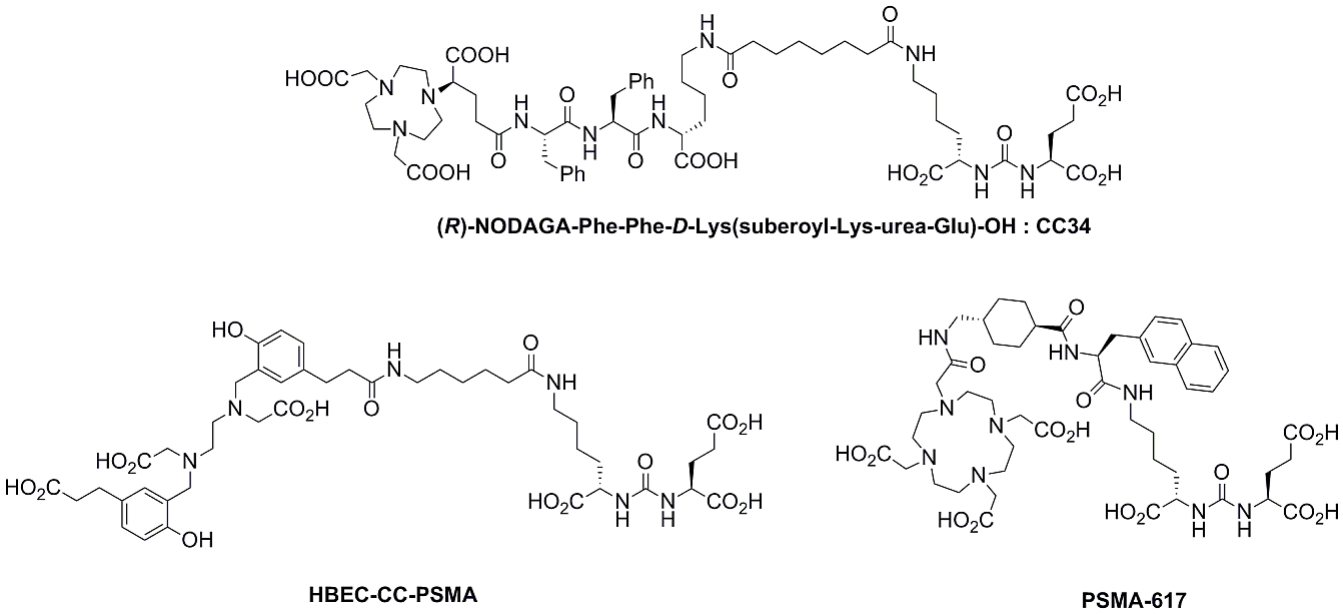
Schematic structures. (R)-NODAGA-Phe-Phe-D-Lys(suberoyl-Lys-urea-Glu) (CC34), HBED-CC-PSMA and PSMA-617.

Herein, the urea-based PSMA inhibitor derived from the lysine-urea-glutamate peptidomimetic structure, was coupled to the spacer Phe-Phe-D-Lys(suberoyl) and functionalized with the enantiomerically pure prochelator (R)-1-(1-carboxy-3-carbotertbutoxypropyl)-4,7-carbotartbutoxymethyl)-1,4,7-triazacyclononane ((R)-NODAGA(tBu)_3_), to obtain (R)-NODAGA-Phe-Phe-D-Lys(suberoyl)-Lys-urea-Glu (CC34) (Fig 1). CC34 radiolabeled with ^68^Ga, ^64^Cu and ^111^In. The ^68^Ga- ^64^Cu- and ^111^In-conjugates were further evaluated in vitro and in vivo in LNCaP tumor xenografts by biodistribution and PET imaging studies. ^68^Ga-HBED-CC-PSMA and ^111^In-PSMA-617 were also evaluated for comparison (Fig 1).

## Material and Methods

The supplier information for all reagents and details of instruments used are provided in the S1 Appendix.

### Synthesis of the urea-based compound CC34

The PSMA-based inhibitor, CC34, was synthesized using standard Fmoc chemistry. Description of the synthesis is given in the S2 Appendix.

### Radiochemistry / Lipophilicity

For the evaluation of the ^68^Ga-labeling efficiency, ^68^Ga-CC34 and ^68^Ga-HBED-PSMA, were prepared within 10 min at 95°C, using the Modular-Lab PharmTracer module by Eckert & Ziegler (Berlin, Germany) (S3 Appendix).

The ^64^Cu- and ^111^In-labeled radiotracers were prepared within 30 min at 95°C and were used without any further purification (S3 Appendix).

The lipophilicity (LogD, pH 7.4) was estimated by the “shake-flask” method (S3 Appendix).

### Saturation binding / Internalization studies

For receptor saturation analysis, PSMA positive LNCaP cells (metastatic lesion of human prostatic adenocarcinoma, ATCC) were seeded at a density of 0.8–1 million cells per well in 6-well poly-L-lysine PLL-coated plates and incubated overnight with medium (RPMI Medium 1640—GlutaMAX containing 1% FBS, 100 U/mL penicillin and 100 μg/mL streptomycin, sodium-pyruvat 1 mM). The next day, the medium was removed, the cells washed and incubated for 1 h at 37°C with fresh binding buffer (RPMI Medium 1640—GlutaMAX containing 1% FBS, 100 U/mL penicillin and 100 μg/mL streptomycin, 50 mM Hepes, 50 μg/mL bacitracin, 0.5% BSA). Afterwards, the plates were placed on ice for 30 min followed by incubation with increasing concentrations of either ^68/nat^Ga-CC34, ^64/nat^Cu-CC34, ^111/nat^In-CC34 and ^111/nat^In-PSMA-617 (1–100 nM) in phosphate-buffered saline binding buffer pH 7.4. After the addition of the radioligands, the cells were incubated for 120 min at 4°C. Non-specific binding was determined in the presence of 2-(phosphonomethyl)-pentanedioic acid (PMPA) at a final concentration of 1 μM. The cells were washed twice with ice-cold PBS, followed by solubilization with 1 N NaOH and the cell-associated radioactivity was measured using a gamma-counter. Specific binding was plotted against the total molar concentration of the added radiotracer. The Kd values and the concentration of the radiotracers required to saturate the receptors (Bmax) were determined by nonlinear regression using GraphPad (Prism 5 Graph Pad Software, San Diego, CA). For all the cell studies the values are normalized for 1x10^6^ cells per well and all data are from two independent experiments with triplicates in each experiment.

For internalization experiments, LNCaP cells were seeded into 6-well plates and treated as described above. Approximately 0.25 pmol of the radiopeptides (^64^Cu-CC34, ^68^Ga-CC34, ^111^In-CC34, ^68^Ga-HBED-CC-PSMA and ^111^In-PSMA-617), were added to the binding buffer and the cells were incubated (in triplicates) for 0.5, 1, 2, 4 and 6 h at 37°C, 5% CO_2_ in case of ^64^Cu-CC34, ^111^In-CC34 and ^111^In-PSMA-617 and for 10, 30, 60, 90, 120 and 180 min at 37°C, 5% CO_2_ in case of ^68^Ga-CC34 and ^68^Ga-HBED-CC-PSMA. To determine nonspecific membrane binding and internalization, excess of PMPA (final concentration 1 μM) was added to selected wells. At each time point, the internalization was stopped by removing the medium and washing the cells twice with ice-cold PBS. To remove the receptor-bound radioligand, an acid wash was carried out twice with a 0.1 M glycine buffer pH 2.8 for 5 min on ice. Finally, cells were solubilized with 1 N NaOH. The radioactivity of the culture medium, the receptor-bound, and the internalized fractions were measured in a γ-counter.

### Metabolic studies

Normal female athymic Balb/c nude mice (2) were injected with approximately 80 pmol / 9 MBq of ^68^Ga-CC34 in a total volume of 0.1 mL of PBS and sacrificed 15 min p.i.. Blood was collected in heparinized tubes and centrifuged (5 min, 1,700g) for plasma isolation. Sample of plasma (300 mL) was transferred to an ultrafiltration device (Vivacon 500; 30,000 molecular weight cutoff [Sartorius Stedium Biotech GmbH]), followed by centrifugation (10 min, 9,660g) for the separation of proteins. Samples from the ultrafiltrate and ^68^Ga-CC34 solution were analyzed by Reversed-Phase High-Performance Liquid Chromatography (RP-HPLC).

### Cell line / Animal model

The PSMA^+^ LNCaP cell line was cultured at 37°C and 5% CO_2_ in (RPMI Medium 1640-GlutaMAX containing 1% FBS, 100 U/mL penicillin and 100 μg/mL streptomycin, sodium-pyruvate 1 mM.

Female athymic Balb/C nude mice (age: 4–6 weeks, weight: 17–20 g) were purchased from Janvier, France. For implantation, the tumor cells were harvested by trypsinization and 5x10^6^ cells in 50% Matrigel (Corning Matrigel Matrix, 354230) and were inoculated subcutaneously into the right shoulder of the mice. After an average of four weeks, tumor size reached 200 to 300 mg and the animals were used for biodistribution and PET imaging studies. All animal experiments were conducted in strict accordance with the recommendations in the Guide for the Care and Use of Laboratory Animals of the National Institutes of Health. The protocol was approved by the Committee on the Ethics of Animals Experiments of the University Medical Center of Freiburg, Germany (Permit Number: G-13/30).

### Biodistribution

Ten pmol of ^68^Ga-CC34 or ^68^Ga-HBED-CC-PSMA (0.7–0.8 MBq / 100 μL), ^64^Cu-CC34 (0.2–0.3 MBq / 100 μL), ^111^In-CC34 or ^111^In-PSMA-617 (0.09–0.1 MBq / 100 μL) in 100 μL. NaCl 0.9% were injected intravenously into the tail vein of LNCaP mice. Animals were sacrificed by isoflurane anesthesia at 1 and 2 h after injection of the ^68^Ga-labeled radiovectors and at 1, 4, 24 and 48 h after the injection of the ^64^Cu- and ^111^In-labeled radiovectors.

The organs of interest were dissected and weighted, and the radioactivity in tissue samples was counted in a γ-counter. Biodistribution data are given as percent of injected activity per gram of tissue (% IA/g) and are means ± SD (n = 4).

To demonstrate the specificity of binding, LNCaP mice were co-injected with 10 pmol of the radiotracers along with 20 nmol of PMPA. Animals were sacrificed at 1 h after injection by isoflurane anesthesia.

### PET studies in LNCaP xenografts

For the ^68^Ga-labeled compounds static images were acquired for a time period of 15 min at 1 and 2 h p.i., and for ^64^Cu-CC34 at 1, 4, 24 and 48 h p.i. PET blocking studies of the ^68^Ga- and ^64^Cu-labeled radioconjougates, were performed as described above and static scans were obtained as previously described. PET-images were corrected for ^68^Ga or ^64^Cu decay and reconstructed with an ordered-subset expectation maximization algorithm provided by the manufacturer. PET images generated by the AMIDE software.

Tracer uptake is expressed as percentage of decay-corrected %IA/g, with a color scale set from 0% to 250% for qualitative comparison among the images.

### Statistical Analysis

All data are expressed as the mean of values±standard deviation (mean±SD). Prism 5 Software (GraphPad Software) was used to determine statistical significance at the 95% confidence level, with a P value of less than 0.05 being considered significantly different.

## Results

### Chemistry

(R)-NODAGA-Phe-Phe-D-Lys was assembled on solid phase and, after cleavage and deprotection, coupled to tBuO-(NHS-suberoyl)Lys-urea-Glu(OtBu)-OtBu. CC34 was isolated after trifluoroacetic acid (TFA) deprotection and RP-HPLC purification with an overall yield of 22% and a purity >99%, as determined by analytical RP-HPLC. Compound identity was confirmed by electrospray ionization-high-resolution mass spectrometry (ESI-HRMS): m/z = 1255.60924 [M+H]^+^, calculated for C_59_H_87_N_10_O_20_ 1255.60926 (S1 Fig).

### Radiochemistry / Lipophilicity

The PSMA urea-based conjugates were labeled with ^68^Ga, ^64^Cu and ^111^In with a labeling yield >98%. The specific activities were ranging between 75 and 80 MBq/nmol for the ^68^Ga-labeled tracers, approximately 40 MBq/nmol for ^64^Cu-CC34 and about 10 MBq/nmol for the ^111^In-labeled radioligands.

With a LogD_octanol/PBS_ of -4.06 ± 0.10 and -4.06 ± 0.06 ^68^Ga-HBED-CC-PSMA and ^111^In-PSMA-617, respectively, show high hydrophilicity, as compared with ^68^Ga-CC34, ^64^Cu-CC34 and ^111^In-CC34, which showed LogD_octanol/PBS_ of -3.54 ± 0.06, -3.01 ± 0.06 and -3.32 ± 0.05, respectively.

### Saturation binding / Internalization studies—Metabolic stability

**Fig 2:**
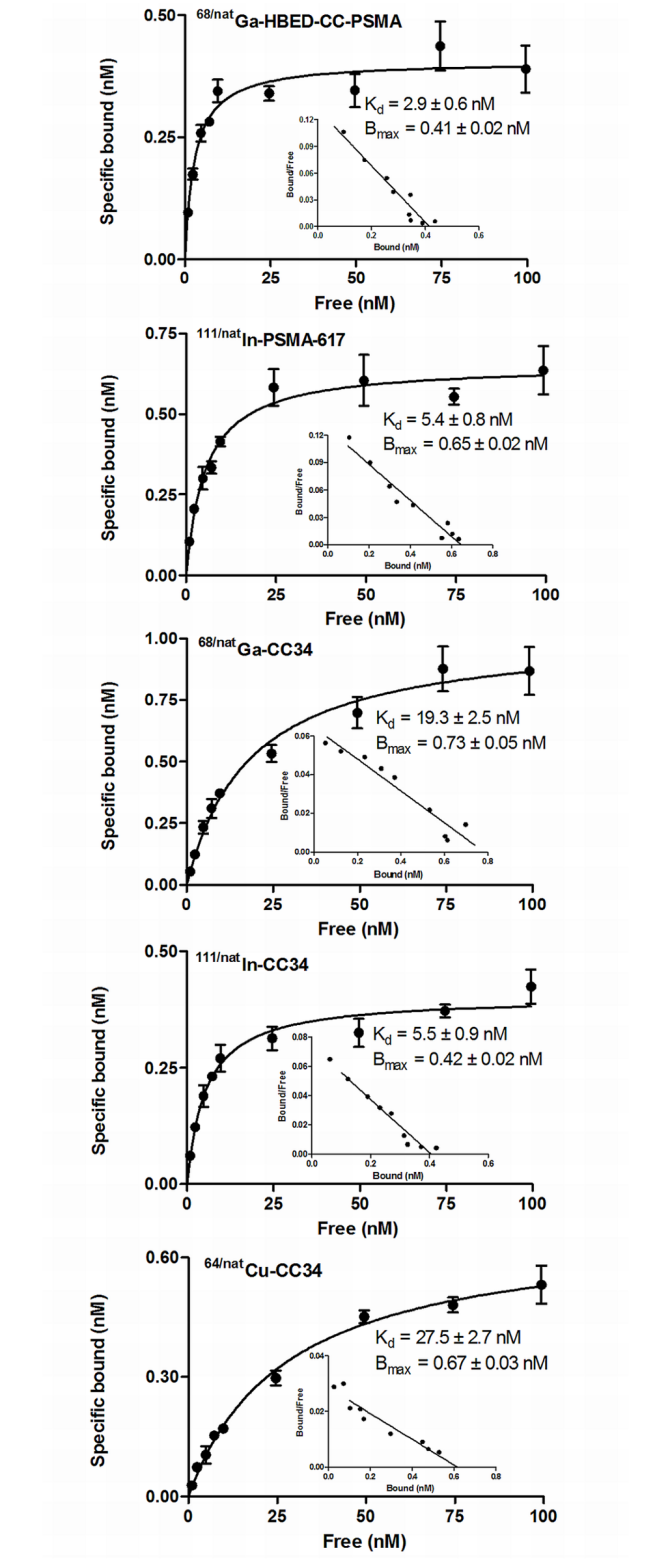
Saturation binding study on intact LNCaP cells. Increasing concentrations of ^68/nat^Ga-HBED-CC-PSMA, ^111/nat^In-PSMA-617, ^68/nat^Ga-CC34, ^111/nat^In-CC34 and ^64/nat^Cu-CC34 were used, ranging from 0.1 to 1,000 nM. All radiotracers exhibited high affinity for the PSMA+ LNCaP cells. ^111/nat^In-CC34 and ^111/nat^In-PSMA-617 are more affine to PSMA Dissociation constant (Kd) and maximum number of binding sites (Bmax) were calculated from nonlinear regression analysis using GraphPad Prism.

All radiotracers exhibited high affinity for the PSMA^+^ LNCaP cells (Fig 2). ^68/nat^Ga-CC34, and ^64/nat^Cu-CC34 have similar affinity with K_d_ values of 19.3 ± 2.5 nM and 27.5 ± 2.7 nM, respectively. Both ^111^In-labeled PSMA-ligands, ^111/nat^In-CC34 and ^111/nat^In-PSMA-617, are more affine to PSMA with K_d_ values of approximately 5 nM. The B_max_ values were also at the same level for all the radioconjugates (approximately 0.6 nM).

**Fig 3:**
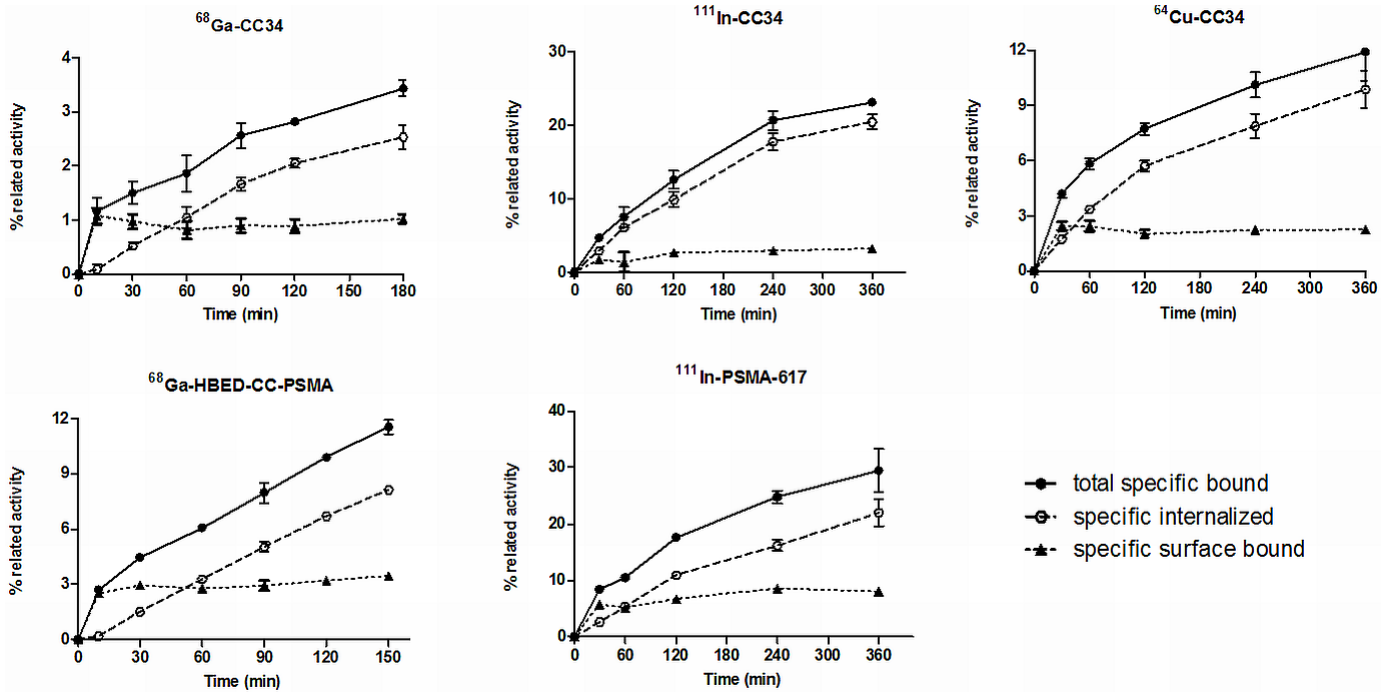
Internalization studies after incubation with LNCaP cells at 37°C. Approximately 75% of the total cell associated activity internalized after 3 h of incubation with the LNCaP cells Cell uptake calculated as cell surface-bound and internalized fraction. Surface bound and receptor-specific internalization expressed as percentage of the applied radioactivity. Nonspecific binding was determined in the presence of 1 μM PMPA.

Internalization studies showed specific internalization at 37°C and the internalized activity always exceeded the surface bound activity (Fig 3). Approximately 75% of the total cell associated activity was internalized after 3 h of incubation.

**Fig 4:**
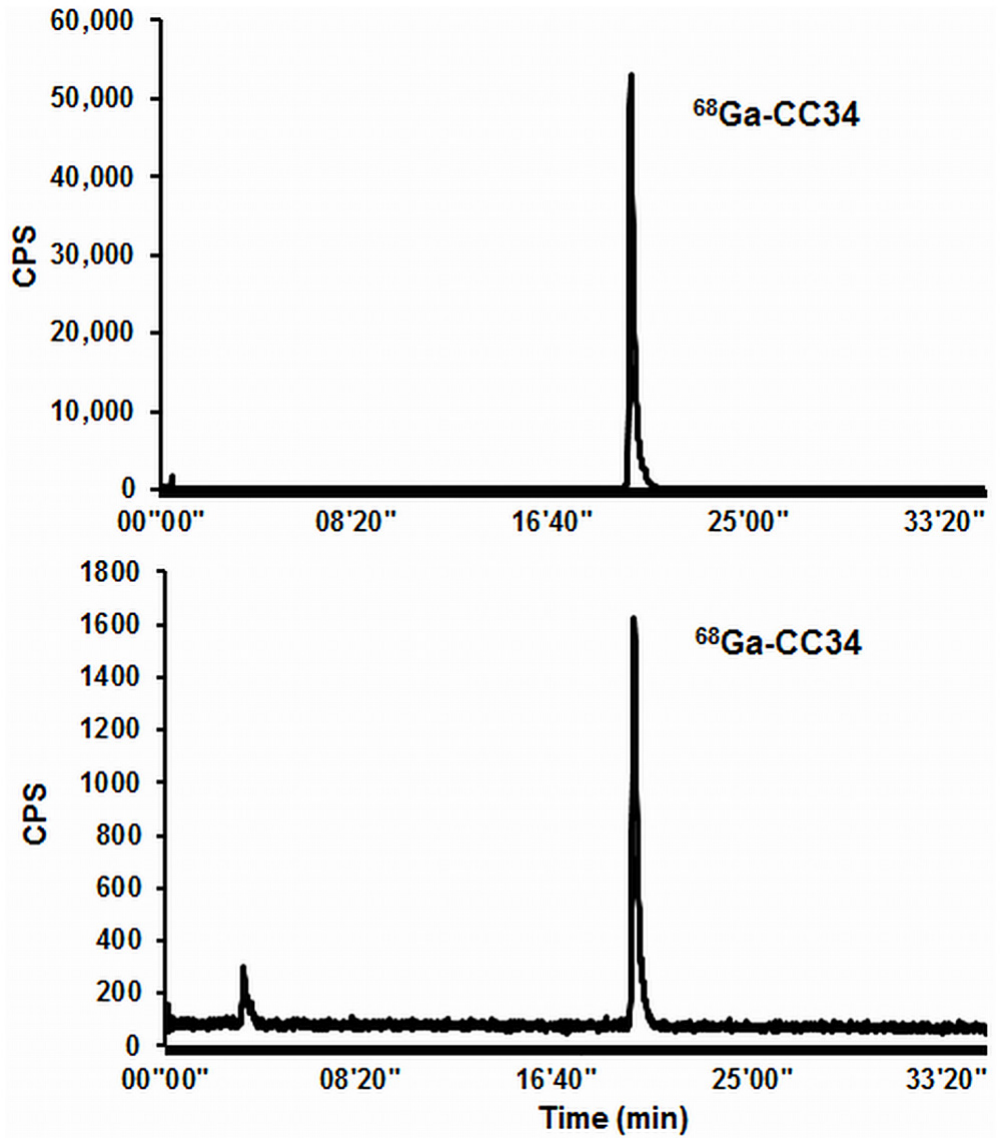
HPLC profiles. A: formulated ^68^Ga-CC34, B: mouse plasma extracted from Balb/c nude mice 15 min after injection of ^68^Ga-CC34.

As demonstrated by RP-HPLC metabolite analysis of plasma samples 15 min p.i. of ^68^Ga-CC34, the remaining circulating activity exclusively consists of intact radiotracer (>95%) (Fig 4).

### Biodistribution in LNCaP xenografts

The biodistribution data are summarized in Tables 1 to 4. ^68^Ga-HBED-CC-PSMA, ^68^Ga- and ^64^Cu-CC34, as well as ^111^In-PSMA-617 displayed rapid blood clearance with approximately 0.2% IA/g left in blood at 2 h p.i.. ^111^In-CC34 exhibited high blood values at early time points (8.1±0.3% IA/g and 2.0±0.8% IA/g at 1 and 2 h p.i. respectively) and slow clearance with about 0.2% IA/g remaining in blood 24 h p.i. Organs such as kidneys, spleen, lung and adrenals with elevated PSMA expression showed high and receptor mediated uptake as shown after the coinjection of 20 nmol of PMPA. ^68^Ga-HBED-CC-PSMA and ^68^Ga-CC34 exhibited similar biodistribution profile with tumor uptake of 15.8±1.4% IA/g and 14.5±2.9% IA/g at 1 h p.i. (*P* = 0.67). Their tumor-to-normal tissue ratios at 1 and 2 h p.i were also comparable (*P*>0.05). The pharmacokinetic performance of ^64^Cu-CC34, at later time points showed faster wash out from the PSMA positive organs compared to the tumor leading to improved tumor to background ratios over time. Both ^111^In-labeled tracers showed the highest tumor uptake compared to the ^68^Ga- and ^64^Cu-labeled tracers (28.5±2.6% IA/g for^111^In-CC34 and 52.1±6.5% IA/g for ^111^In-PSMA-617 at 1 h p.i.) which dropped to 16.5±1.9% IA/g and 21.2±1.6% IA/g at 48 h p.i., respectively. Compared to ^64^Cu-CC34 and ^111^In-PSMA-617, ^111^In-CC34 exhibited high and retained kidney uptake over time.

**Table 1:** Biodistribution data of ^68^Ga-HBED-CC-PSMA and ^68^Ga-CC34 in LNCaP.

	^68^Ga-HBED-CC-PSMA			^68^Ga-CC34		
Organ ^a^	1 h	1 h blocked	2 h	1 h	1 h blocked	2 h
Blood	0.2±0.04	0.4±0.04	0.2±0.04	0.5±0.3	0.3±0.4	0.2±0.1
Heart	0.6±0.06	0.2±0.06	0.6±0.02	0.4±0.2	0.1±0.06	0.3±0.03
Liver	0.4±0.01	0.4±0.05	0.4±0.05	0.3±0.1	0.3±0.2	0.2±0.1
Spleen	31±6	2.1±0.06	29±2	15±2	0.4±0.02	9.5±3.4
Lung	3.5±0.2	0.7±0.2	3.2±0.2	1.8±0.6	0.3±0.1	1.3±0.1
Kidney	208±4	11±3	211±29	263±29	6.4±0.2	271±36
Stomach	0.7±0.1	0.3±0.01	1±0.6	0.9±0.2	0.2±0.1	0.8±0.1
Intestine	0.5±0.1	0.3±0.04	0.5±0.2	1.2±0.4	0.2±0.1	0.8±0.3
Adrenal	23±4	1.1±0.5	23±0.8	11.7±1.4	0.2±0.1	12±1.3
Pancreas	1.4±0.3	0.3±0.04	2±0.5	0.9±0.1	0.1±0.02	0.8±0.4
Muscle	0.6±0.06	0.2±0.1	0.6±0.2	0.4±0.08	0.1±0.04	0.2±0.1
Bone	0.5±0.05	0.4±0.2	0.4±0.2	0.7±0.1	0.4±0.3	0.8±0.2
LNCaP-tumor	15.8±1.4	4.1±1.2	14.8±2.2	14.5±2.9	1.2±0.03	11.2±2.3
Tumor/blood	68±4		83±15	31±13		53±10
Tumor/kidney	0.08±0.01		0.07±0.02	0.06±0.01		0.04±0.01
Tumor/muscle	29±6		24±3	36±6		47±14

Biodistribution data at 1 and 2 h along with blocking studies at 1 h after the injection of 10 pmol of ^68^Ga-CC34 or ^68^Ga-HBED-CC-PSMA (0.7–0.8 MBq / 100 μL) into the tail vein of LNCaP mice.

**Table 2:** Biodistribution data of ^64^Cu-CC34.

Organ ^a^	1 h	1 h blocked	4 h	24 h	48 h
Blood	0.5±0.08	0.1±0.03	0.2±0.03	0.1±0.02	0.06±0.00
Heart	0.7±0.3	0.2±0.01	0.3±0.02	0.2±0.1	0.2±0.01
Liver	0.7±0.06	0.6±0.01	0.6±0.1	0.5±0.1	0.4±0.01
Spleen	31±2.9	0.7±0.5	6.7±1.2	0.6±0.1	0.2±0.05
Lung	2.3±0.2	0.7±0.2	1.3±0.2	0.3±0.1	0.2±0.04
Kidney	275±26	8±0.8	275±21	22±2	2.0±1.2
Stomach	0.9±0.1	0.4±0.3	0.9±0.1	0.3±0.1	0.2±0.02
Intestine	0.8±0.3	0.3±0.1	0.9±0.4	0.3±0.1	0.2±0.03
Adrenal	11.0±2.2	0.6±0.3	6.2±1.0	1.0±0.4	0.2±0.05
Pancreas	1.4±0.4	0.1±0.002	0.6±0.2	0.1±0.03	0.06±0.00
Muscle	0.4±0.07	0.06±0.02	0.2±0.1	0.07±0.02	0.03±0.01
Bone	0.3±0.08	0.06±0.02	0.3±0.2	0.2±0.1	0.2±0.07
LNCaP-tumor	20.3±1.5	1.8±0.5	19.1±2.1	7.1±1.1	3.2±0.2
Tumor/blood	41±10		114±17	81±6	58±4
Tumor/kidney	0.07±0.002		0.07±0.009	0.3±0.06	2±0.8
Tumor/muscle	61±18		103±21	112±60	118±47

Biodistribution data at 1, 4, 24 and 48 h along with blocking studies at 1 h after the injection of 10 pmol of ^64^Cu-CC34 (0.2–0.3 MBq / 100 μL) into the tail vein of LNCaP mice.

**Table 3:** Biodistribution data of ^111^In-CC34.

Organ ^a^	1 h	1 h blocked	4 h	24 h	48 h
Blood	8.1±0.3	13.8±0.9	2.0±0.8	0.2±0.02	0.1±0.03
Heart	2.8±0.34	3.6±0.3	1.0±0.2	0.1±0.02	0.1±0.01
Liver	3.2±0.5	5.1±0.6	1.1±0.3	0.3±0.02	0.2±0.05
Spleen	38±5	3.7±0.8	34.1±7.4	4.5±0.9	2.5±0.9
Lung	7.6±0.7	8.2±1.3	4.9±1.4	1.2±0.3	0.5±0.03
Kidney	148±14	16.9±1.6	256±29	210±33	128±11
Stomach	1.6±0.1	1.9±0.2	0.9±0.1	0.4±0.1	0.2±0.1
Intestine	2.1±0.5	3.4±0.5	0.6±0.1	0.1±0.02	0.1±0.01
Adrenal	12.9±2.7	3.4±1.5	15.8±1.0	4.6±0.9	2.8±0.1
Pancreas	2.6±0.3	1.9±0.2	1.5±0.3	0.4±0.1	0.2±0.1
Muscle	1.5±0.1	1.6±0.1	0.6±0.02	0.1±0.03	0.1±0.02
Bone	1.2±0.2	1.3±0.1	0.5±0.2	0.2±0.1	0.3±0.1
LNCaP-tumor	28.5±2.6	5.3±0.8	31.3±5.9	21.4±3.7	16.5±1.9
Tumor/blood	3.4±0.5		16±3	119±31	127±11
Tumor/kidney	0.2±0.02		0.1±0.03	0.1±0.01	0.1±0.01
Tumor/muscle	19±1		52±8	186±35	226±37

Biodistribution data at 1, 4, 24 and 48 h along with blocking studies at 1 h after the injection of 10 pmol of ^111^In-CC34 (0.09–0.1 MBq / 100 μL) into the tail vein of LNCaP mice.

**Table 4:** Biodistribution data of ^111^In-PSMA-617.

Organ ^a^	1 h	1 h blocked	4 h	24 h	48 h
Blood	0.6±0.2	0.4±0.1	0.1±0.02	0.01±0.0	0.01±0.0
Heart	0.5±0.2	0.2±0.1	0.07±0.01	0.01±0.01	0.02±0.01
Liver	0.4±0.1	0.3±0.03	0.1±0.01	0.06±0.01	0.04±0.0
Spleen	29±5	0.5±0.1	2.1±0.6	0.1±0.05	0.1±0.01
Lung	2.6±0.7	0.7±0.1	0.5±0.04	0.2±0.1	0.1±0.02
Kidney	290±29	4.1±0.6	126±15	3.0±0.5	1.3± 0.1
Stomach	0.9±0.2	0.2±0.05	0.3±0.1	0.03±0.0	0.03±0.01
Intestine	1.0±0.7	0.3±0.05	0.2±0.1	0.02±0.0	0.02±0.01
Adrenal	8.0 ±0.4	0.3±0.2	2.8±0.6	0.01±0.01	0.1±0.01
Pancreas	1.3±0.1	0.2±0.03	0.3±0.1	0.02± 0.0	0.02±0.01
Muscle	0.4±0.1	0.07±0.02	0.1±0.02	0.01± 0.0	0.01±0.00
Bone	0.8±0.3	0.1±0.06	0.3± 0.1	0.2± 0.04	0.1±0.06
LNCaP-tumor	52.1±6.5	4.8±0.6	51.4±4.5	27.2±1.9	21.2±1.6
Tumor/blood	72±19		772±153	4768±110	5343±1033
Tumor/kidney	0.2±0.01		0.4±0.04	9±2	16±2
Tumor/muscle	148±61		582±24	2819±864	1987±177

Biodistribution data at 1, 4, 24 and 48 h along with blocking studies at 1 h after the injection of 10 pmol of ^111^In-PSMA-617 (0.09–0.1 MBq / 100 μL) into the tail vein of LNCaP mice.

^a^ Data are expressed in percentage of injected activity per gram of tissue (%IA/g) and are presents as mean ± SD (n = 3–4). This applies for Tables 1 to 4.

PSMA tumor binding specificity was demonstrated by a reduction of the tumor uptake of about 75% in case of ^68^Ga-HBED-CC-PSMA and ^111^In-CC34 and more than 90% for ^68^Ga-CC34, ^64^Cu-CC34 and ^111^In-PSMA-617.

### Small-animal PET studies

**Fig 5:**
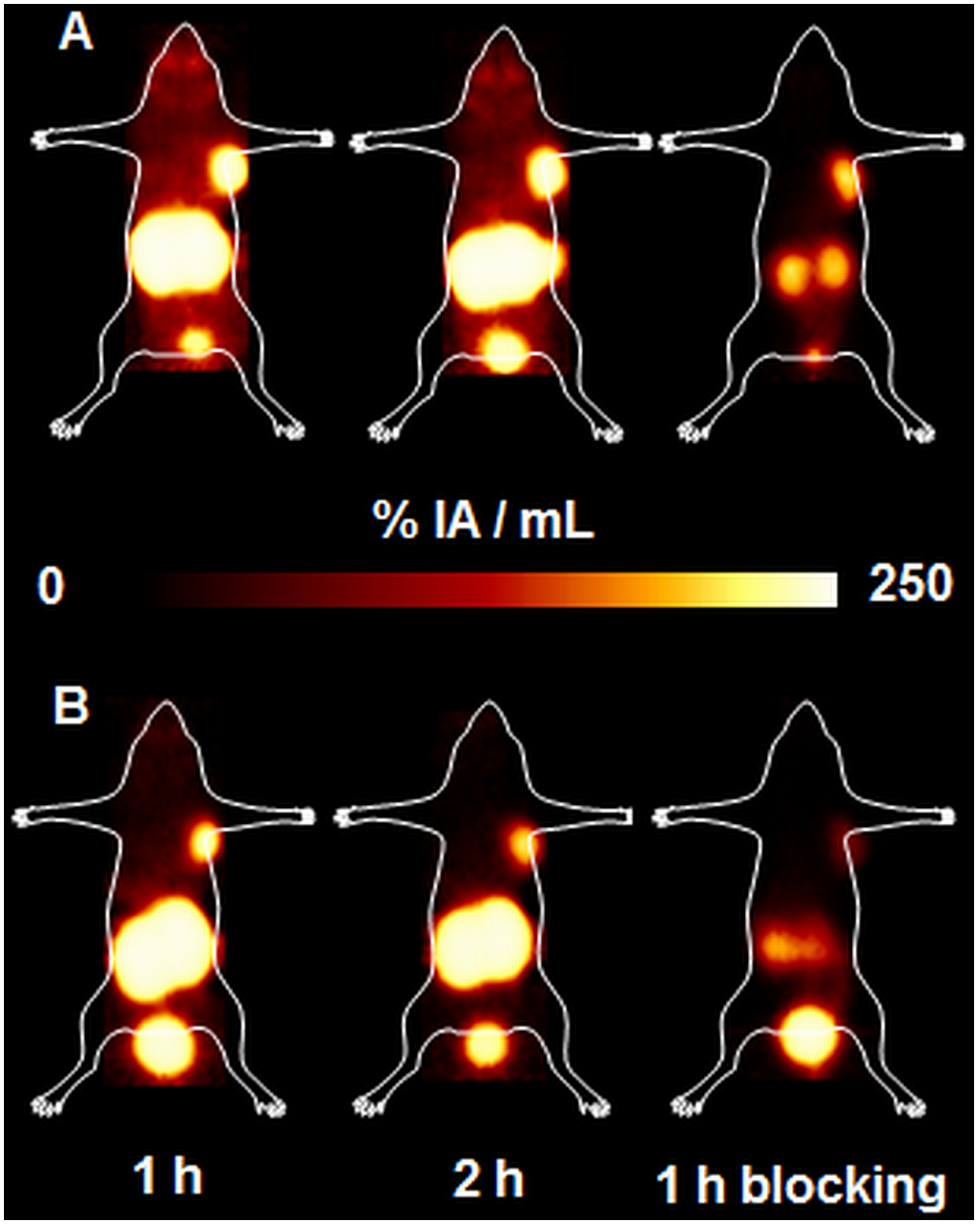
Maximum intensity projections (MIPs) PET images. (A) ^68^Ga-HBED-CC-PSMA and (B) ^68^Ga-CC34 upon their injection in LNCaP tumor bearing mice at 1 h and 2 h p.i along with blocking studies at 1h p.i.. The images clearly visualized the tumors. They showed high kidney uptake at early time points and prove the specificity of the radiopeptides for the PSMA receptors, as the tumor is hardly visualized after co-injection of excess of PMPA.

Representative PET images obtained upon injection of ^68^Ga-HBED-CC-PSMA and ^68^Ga-CC34 in LNCaP xenografts at 1 and 2 h p.i. (Fig 5), with specific tumor and kidney uptake, as shown by the blocking studies at 1 h p.i..

**Fig 6:**
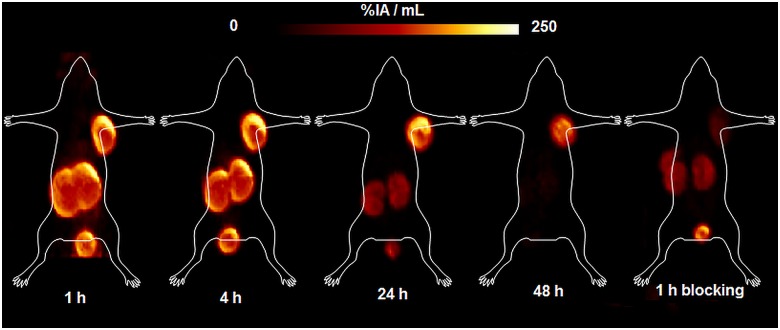
Maximum intensity projections (MIPs) PET images of ^64^Cu-CC34. The images were acquired upon the injection of ^64^Cu-CC34 on LNCaP tumor bearing mice at 1, 4, 24 and 48 h p.i along with blocking studies at 1h p.i.. They showed good pharmacokinetics, with clear deliniation of the tumor, gradual reduction of the kidney uptake at 24 and 48 h, respectively. The ^64^Cu-labeled conjugate showed low liver uptake.

The pharmacokinetics of ^64^Cu-CC34 was also determined by small-animal PET (Fig 6) at 1, 4, 24 and 48 h in the same animals. The radiotracer is specifically taken up by the PSMA positive organs at early time points. The PET studies of ^64^Cu-CC34 showed good pharmacokinetics, with gradually reduced uptake in kidneys at 24 and 48 h, respectively. The ^64^Cu-labeled conjugate showed low liver uptake.

## Discussion

Prostate cancer is a complex and biologically heterogeneous disease and therefore cannot be fully assessed with conventional imaging alone. Radionuclide molecular imaging with positron emission tomography (PET) is poised to fill this unmet need through noninvasive detection of the multiple molecular and cellular processes that are active in prostate cancer patients [30].

PSMA is primarily expressed in the human prostate epithelium, salivary and lacrimal glands as well as kidneys with enhanced expression by almost all prostate cancers and further up-regulation in poorly differentiated, metastatic and hormone-refractory carcinomas [31]. These characteristics render PSMA as promising target for prostate cancer imaging and potential therapy. Many efforts have been made especially during the last decade in regard to the development of PSMA-based imaging agents with particular focus on the low molecular weight PSMA inhibitors. A variety of chelators and spacers were introduced to the urea-based PSMA inhibitors and the derived conjugates were labeled with a series of radionuclides for SPECT and PET imaging [17–29]. A representative example of this class of radiotracers is the peptidomimetic structure Lys-NH-CO-NH-Glu when coupled to the spacer 6-amino-hexanoic acid (Ahx) and functionalized with the chelator *N*,*N′*-dis [2-hydroxy-5-(carboxyethyl)benzyl] ethylenediamine-*N*,*N′*–diacetic acid (HBED-CC) to obtain HBED-CC-Ahx-Lys-NH-CO-NH-Glu (HBED-CC-PSMA). The ability to image PSMA using ^68^Ga-HBED-CC-PSMA shows great promise preclinically and clinically [25,32].

Our goal was to develop and evaluate a versatile probe suitable for imaging of PSMA-positive tumors. Thus, (R)-NODAGA(tBu)_3_ was conjugated to the well-established Lys-urea-Glu PSMA-inhibitor, through a Phe-Phe-*D*-Lys(suberoyl) linker [17,18,20,28]. Because of the small volume of the triazacyclononane cage and its ability to coordinate metal ions, NODAGA is particularly attractive for the rapid and stable chelation of ^111^In for SPECT, as well as ^68^Ga and ^64^Cu applied in PET imaging. The choice of the Phe-Phe-Lys(suberoyl) linker has already proven to be successful by Banerjee et al. [28].

CC34 was labeled with ^68^Ga, ^64^Cu and ^111^In, to be used for PET and SPECT imaging and intraoperative applications. Due to its availability from generator systems, the positron emitter ^68^Ga has gained rapidly increasing interest in the field of radiopharmaceutical chemistry [33]. ^64^Cu is a positron emitter with a longer half-life (12.7 h) compared to ^68^Ga (67.8 min) and can give PET-images at later time points with improved tumor-to-background ratios [34]. ^64^Cu-radiopharmaceuticals can be produced centrally and shipped to distant hospitals. ^111^In is used as an important SPECT label. Additionally, intraoperative gamma probes are now an important, well-established technology in the management of cancer, particularly in the detection of sentinel lymph nodes.

CC34, being labeled with ^68^Ga, ^64^Cu and ^111^In, showed high affinity towards PSMA on LNCaP cells, with ^111/nat^In-CC34 exhibiting a significantly increased affinity compared to the ^68/nat^Ga- and ^64/nat^Cu-conjugates. In our saturation binding assay, ^68/nat^Ga-HBED-CC-PSMA revealed the highest affinity towards PSMA while ^111/nat^In-PSMA-617 was as affine as ^111/nat^In-CC34.

To figure out if CC34 is subjected to in vivo metabolic degradation and to which extent, RP-HPLC metabolite analysis of plasma samples was performed. Fifteen min p.i. of ^68^Ga-CC34 more than 95% of the remaining circulating activity corresponds to intact radiotracer. When the Lys-NH-CO-NH-Glu peptidomimetic structure was coupled to the spacer Phe-Phe-Lysine-suberoyl (L-amino acid spacer) and functionalized with the chelator (1-(1,3-carboxy-propyl)-4,7,10(carboxymethyl)-1,4,7,10 tetraazacyclo-dodecane (DOTAGA) [23], rapid in vivo metabolization of the ^68^Ga-labeled radiovector was demonstrated. In the same report, the D-amino acids spacer led to an in vivo metabolic stable radiotracer. In our study we proved that only the substitution of L- with D-Lysine in the spacer, resulted in high in vivo stability of ^68^Ga-CC34.

The enhanced PSMA affinity, ^111/nat^In-CC34 and ^111/nat^In-PSMA-617 resulted in two- to five-fold increased internalization rate and higher but also longer tumor retention compared to ^64/nat^Cu- and ^68/nat^Ga-CC34 and ^68/nat^Ga-HBED-CC-PSMA. Although the binding affinity can be considered as the most crucial parameter which greatly influences the tumor uptake, the overall pharmacokinetic performance of a radiotracer is determined by many other factors which certainly need to be taken into consideration. In particular, parameters such as, lipophilicity, charge, plasma protein binding and molecular weight also influence the pharmacokinetic performance of a radiotracer. CC34 can be considered as a strong evidence of the above assumption. ^68^Ga-CC34 and ^64^Cu-CC34 demonstrated fast clearance from blood and normal tissues, even kidneys as proved by biodistribution and PET-studies in the case of the copper-64 labeled compounds. The superiority of NODAGA compared to HBED-CC to be efficiently labeled with ^64^Cu allows for the conduction of biodistribution/imaging studies at later time points and therefore the complete accomplishment of the pharmacokinetic performance of the radiotracer. ^64^Cu-CC34 showed faster wash out from the PSMA positive organs compared to the tumor leading to improved tumor to background ratios. The very low liver uptake of ^64^Cu-CC34 at all time points in combination with the short blood circulation need to be pointed out since this is a strong indication of the excellent in vivo stability of the ^64^Cu-NODAGA complex. Unfortunately, this was not the case for ^111^In-CC34. The affinity of ^111/nat^In-CC34 towards PSMA was higher compared to ^68/nat^Ga-CC34 and ^64/nat^Cu-CC34 by a factor of about 4 and as it was anticipated tumor uptake was higher and longer retention was also observed. Surprisingly, ^111^In-CC34 exhibited long blood circulation and highly retained activity in the kidneys even at 48 h p.i.. Unfortunately, this undesired in vivo profile eliminates its applicability as a SPECT imaging agent. The pharmacokinetics of the alanine-containing DOTA-conjugated PSMA inhibitor, PSMA-617, labeled with ^111^In, on the other hand, was excellent with impressive tumor to background ratios over time. Protein binding studies (data not shown) 15 min p.i. of ^111^In-CC34 and ^111^In-PSMA-617 in mice revealed that 83 and 53%, respectively, of the remaining circulating activity was bound to the proteins. This biodistribution profile of ^111^In-CC34 cannot be explained by its hydrophilicity (LogD_octanol/PBS_ = -3.32). NODAGA, although is not usual, might form a seven-coordinate complex with ^111^In, and if this is the case here, ^111^In-CC34 with an additional charge appears to bind strongly to proteins [35]. The seventh coordination site could be occupied by a Lys side chain of a protein. It is worth noting that ^177^Lu-PSMA-617, exhibited similar pharmacokinetics compared to ^111^In-PSMA-617, which is also characterized by high and retained tumor uptake and almost complete elimination of the radioactivity through kidneys within 24 h [26].

The chelators 1,4,8,11-tetraazabicyclo[6.6.2]-hexadecane-4,11-diacetic acid (CB2-TE2A) and 1,4,7,10-tetraazacyclododecane- *N*,*N′*,*N″*,*N‴*-tetraacetic acid (DOTA) were used for the functionalization of the Phe-Phe-L-Lys(suberoyl-Lys-urea-Glu). Significantly high in vivo stability, as evidenced by the low liver uptake and fast blood clearance was demonstrated for the ^64^Cu-CB2-TE2A-conjugated radiotracer with comparable tumor to blood and tumor to muscles ratios with ^64^Cu-CC34 at 1 h p.i.. High liver uptake and slow blood clearance for the ^64^Cu-DOTA-conjugated radiotracer was indicative of free Cu(II), which is accumulated in liver [29]. Kidney uptake of the ^64^Cu-CB2-TE2A-PSMA-based tracer was two-fold lower compared to ^64^Cu-CC34 and very fast wash out was demonstrated. Although the PSMA-mediated renal uptake has been shown to be specific by us and others, it is interesting that variations are observed not only in regard to the absolute kidney uptake but also as far as the renal elimination concerns.

## Conclusions

Our data document the versatility of the (R)-NODAGA-functionalized PSMA-based radiotracer, CC34, labeled with ^68^Ga and ^64^Cu, as a highly specific targeted PET imaging agent. In particular, the favorable pharmacokinetic performance of the ^64^Cu-labeled radiotracer renders it a promising tracer that would enable sufficient imaging of PSMA positive tissues which are characterized by elevated expression of PSMA or in the case that delayed imaging is required. This tracer is currently prepared for clinical translation.

## Supporting Information

S1 Appendix Reagents and Instrumentation. (DOC)

S2 Appendix Synthesis of the urea-based compound CC34. (DOC)

S3 Appendix Radiochemistry / Lipophilicity. (DOC)

S1 Fig ESI-HRMS analysis of CC34. (DOC)
