# Supplementary material for: (R)-NODAGA-PSMA: A Versatile Precursor for Radiometal Labeling and Nuclear Imaging of PSMA-Positive Tumors
Source: PLoS One. 2015 Dec 23;10(12):e0145755. doi: 10.1371/journal.pone.0145755 (PMC4689406; doi:10.1371/journal.pone.0145755)
Supplement: S1 Appendix — (DOC) [file pone.0145755.s001.doc]

**S1 Appendix: Reagents and Instrumentation**

All reagents were of the best grade available and were purchased from common suppliers. The pro-chelator (R)-NODAGA(tBu)_3_ ester is commercially available from CheMatech, Dijon, France. HBED-CC-PSMA and PSMA-617 were commercially available from ABX GmbH, Germany. ^68^Ge/^68^Ga-generator IGG100 was available from Eckert & Ziegler (Berlin, Germany). ^64^CuCl_2_ was purchased from Acom (Italy). All culture reagents were from Gibco BRL, Life Technologies (Grand Island, NY).

Compounds were purified by semi-preparative RP-HPLC on a Dionex Ultimate 3000 system (Thermo Scientific) equipped with a Nucleodur C18ec column (Macherey-Nagel, 250 × 10 mm, 5 μm) with a gradient program from 15% to 45% of solvent B over 40 min (solvent A is water with 0.1% TFA and solvent B is acetonitrile with 0.1% TFA) at a flow rate of 3 mL/min. Purity was determined by RP-HPLC on a Dionex Ultimate 3000 system equipped with a photodiode array detector, on a Kinetex C18 column (Phenomenex, 50 × 2.1 mm, 2.6 μm), at a flow rate of 0.5 mL/min and with a gradient program from 5% to 100% of solvent B over 5 min. Low-resolution mass spectra (MS-ESI) were obtained on Amazon SL spectrometer (Brucker). The structure of the final compound was confirmed by high resolution electrospray mass spectrometry (HRMS-ESI), carried out using a LTQ-Orbitrap XL (Thermo Scientific).

The quality control of the radiolabeled compounds was performed by analytical RP-HPLC on an analytical 120-5 C18 Nucleosil column (250 x 4.5 mm) applying a linear gradient of 15-90% solvent B in 25 min at a flow rate of 1 mL/min. (solvent A, 0.1% TFA/H_2_O; solvent B, .1%TFA/Acetonitrile). Ultraviolet detection was performed using a Knauer detector at 280 nm. For radioactivity measurement, a Na(TI) well-type scintillation Gina star was used. The radiotracer solutions were prepared by dilution with 0.9% NaCl.

Quantitative γ-counting was performed with a COBRA 5003 γ-system well counter from Packard Instrument (USA). All experiments were carried out 2 times in triplicate.

For PET studies a dedicated small-animal PET scanner (Focus 120 microPET scanner; Concorde Microsystems Inc.) was used.
