# Supplementary material for: (R)-NODAGA-PSMA: A Versatile Precursor for Radiometal Labeling and Nuclear Imaging of PSMA-Positive Tumors
Source: PLoS One. 2015 Dec 23;10(12):e0145755. doi: 10.1371/journal.pone.0145755 (PMC4689406; doi:10.1371/journal.pone.0145755)
Supplement: S2 Appendix — (DOC) [file pone.0145755.s002.doc]

**S2 Appendix: Synthesis of the urea-based compound CC34**

Fmoc-*D*-Lys-Wang resin (0.60 mmol/g, 0.25 mmol) was allowed to swell in DMF (5 mL) for 30 min at room temperature in a syringe equipped with a polyethylene frit. The solvent was removed and a solution of 20% piperidine in DMF (5 mL) was added to the resin. The mixture was shaken at RT for 10 min. The deprotection treatment was repeated twice, and the resin as washed with DMF (4 x 8 mL). Fmoc-*L*-Phe-OH (3 equiv.) was pre-activated with HBTU (3 equiv.) and DIPEA (3 equiv.) in DMF (3 mL) for 5 min and the mixture was added to the resin. Coupling was performed over 1 h at RT under gentle shaking and its completeness was confirmed by a TNBS assay. The aforementioned sequence was repeated to introduce the second phenylalanine. Then (R)-NODAGA(tBu)_3_ (2.3 equiv.) was coupled to the peptidyl-resin in the presence of HATU (2.3 equiv.) and DIPEA (2.3 equiv) in DMF (5 mL) over 15 h at RT and the resin was extensively washed with DMF, DCM and diethylether and dried under vacuum. Cleavage and deprotection of the peptide were performed by treating the resin with a solution of TFA/TIPS/water (95:2.5: 2.5) at RT. A prolonged reaction time (6 h) was required to obtain the complete hydrolysis of the tBu ester groups of (R)-NODAGA. The filtrate from the cleavage mixture was concentrated, precipitated in cold Et_2_O and collected by centrifugation (twice), and lyophilized to afford crude peptide. The product was purified by semi-preparative RP-HPLC to afford (R)-NODAGA-Phe-Phe-D-Lys-OH, 4 TFA as a white powder (155 mg, 49%)

tBuO-(NHS-suberoyl)Lys-urea-Glu(OtBu)-OtBu (22 mg) was dissolved in DMF (2 mL) and (R)-NODAGA-Phe-Phe-D-Lys-OH, 4 TFA (1.5 equiv) and DIPEA (10 equiv.) were added. The solution was stirred during 4 h at RT and then evaporated to dryness under reduced pressure. The hydrolysis of the tBu ester groups was performed in the presence of a solution of TFA/TIPS/water (95.5:2.5:2.5) at RT. After 1 h, the product was concentrated, precipitated in cold Et_2_O and collected by centrifugation (twice). The crude product was purified by RP-HPLC to give CC34 (3 TFA salt) as a white powder (15.6 mg, 46%, purity > 99%). ESI-HRMS m/z calculated for C_59_H_86_N_10_O_20_: 1255.60926 [M+H]^+^, measured: 1255.60968.

**References**

1. Maresca KP, Hillier SM, Femia FL, et al. A series of halogenated heterodimeric inhibitors of prostate-specific membrane antigen (PSMA) as radiolabeled probes for targeting prostate cancer. J Med Chem. 2009;52: 347-357.
2. Banerjee SR, Foss CA, Castanares M, et al. Synthesis and evaluation of technetium-99m and rhenium-labeled inhibitors of the prostate-specific membrane antigen (PSMA). J Med Chem. 2008;51: 4504-4517.
3. Banerjee SR, Pullambhatla M, Byun Y, et al. ^68^Ga-labeled inhibitors of prostate-specific membrane antigen (PSMA) for imaging prostate cancer. J Med Chem. 2010;53: 5333-5341.
