# Supplementary material for: (R)-NODAGA-PSMA: A Versatile Precursor for Radiometal Labeling and Nuclear Imaging of PSMA-Positive Tumors
Source: PLoS One. 2015 Dec 23;10(12):e0145755. doi: 10.1371/journal.pone.0145755 (PMC4689406; doi:10.1371/journal.pone.0145755)
Supplement: S3 Appendix — (DOC) [file pone.0145755.s003.doc]

**S3 Appendix: Radiochemistry / Lipophilicity**

**^68^Ga-CC34 and ^68^Ga-HBED-CC-PSMA:** The ^68^Ge/^68^Ga-generator was eluted with 7 mL HCl 0.1 N and the eluate (~ 450 MBq) was loaded onto a cation exchange column (Strata-XC, Phenomenex). ^68^Ga was eluted with 800 μL of a mixture of acetone/HCl (97.6%/0.02 N) directly in a vial containing 2 mL sodium acetate buffer (0.2 M, pH 4.0), 20 μL ascorbic acid (500 mg / 5 mL) and 10 μg of the conjugates, followed by SepPak C-18 purification to remove uncomplexed radiometal. The radiotracers were analyzed with (analytic) RP-HPLC.

**^64^Cu-CC34:** The ^64^Cu-labeled radiotracer were prepared by dissolving 10 μg (approximately 8 nmol) of CC34 in 250 μL ammonium acetate buffer (0.1 mol/L, pH 8.0), followed by incubation with ^64^CuCl_2_ (180-200 MBq) for 30 min at 95 °C and was used without any further purification step.

**^111^In-CC34 and ^111^In-PSMA-617:** The ^111^In-labeled radiotracers were prepared by dissolving 5 μg (approximately 4 nmol) of CC34 and PSMA-617 in 250 μL ammonium acetate buffer (0.1 mol/L, pH 8.0), followed by incubation with ^111^InCl_3_ (40 MBq) for 30 min at 95 °C and were used without any further purification step.

The radiotracer solutions were prepared by dilution with 0.9% NaCl.

The lipophilicity (LogD, pH 7.4) was estimated by the “shake-flask” method: The labeled conjugates (10 μL of 100 nM, 6.3 MBq / nmol) were added to a solution of 1-octanol (500 μL) and of PBS (500 μL, pH 7.4). The mixture was vortexed for 1 h to reach the equilibrium and then centrifuged (3000 rpm) for 10 min. From each phase, an aliquot (100 μL) was pipetted out and measured in a γ-counter. Each measurement was repeated five times. Care was taken to avoid cross-contamination between the phases. The partition coefficient was calculated as the average log ratio of the radioactivity in the organic fraction and the PBS fraction.
