# Supplementary figures and images for: (R)-NODAGA-PSMA: A Versatile Precursor for Radiometal Labeling and Nuclear Imaging of PSMA-Positive Tumors
Source: PLoS One. 2015 Dec 23;10(12):e0145755. doi: 10.1371/journal.pone.0145755 (PMC4689406; doi:10.1371/journal.pone.0145755)

**
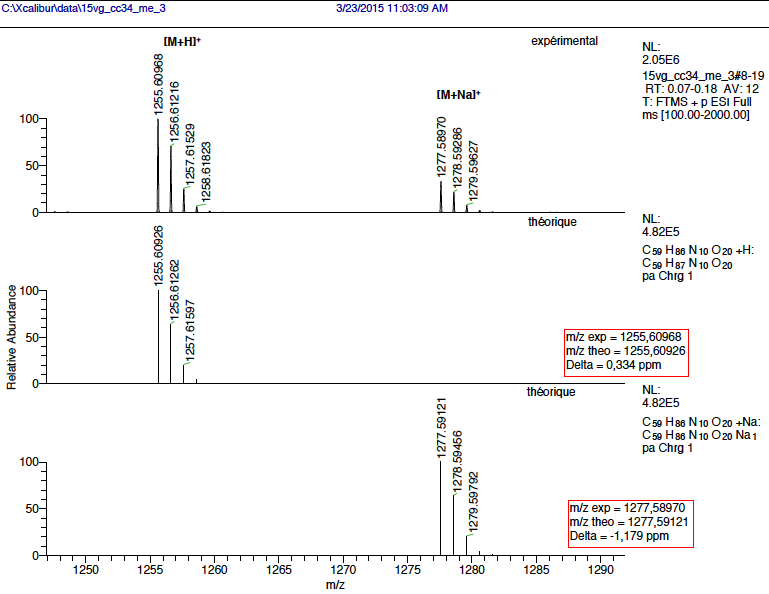
**

**Figure S1 : ESI-HRMS analysis of CC34.**

Supplement: S1 Fig — (DOC) [file pone.0145755.s004.doc]
